# O Estudo ELSA-Brasil e Nossa Deformação Miocárdica

**DOI:** 10.36660/abc.20250265

**Published:** 2025-06-04

**Authors:** Márcio S. M. Lima

**Affiliations:** 1 Hospital das Clínicas Faculdade de Medicina Universidade de São Paulo São Paulo SP Brasil Instituto do Coração do Hospital das Clínicas da Faculdade de Medicina da Universidade de São Paulo, São Paulo, SP – Brasil; 2 Grupo Fleury São Paulo SP Brasil Grupo Fleury, São Paulo, SP – Brasil

**Keywords:** Ecocardiografia, Função Ventricular, Estudos Longitudinais

A análise da deformação miocárdica (strain) utilizando a tecnologia de rastreamento de speckles na ecocardiografia não é mais algo novo e inovador. Vários anos se passaram desde sua validação.^1^ Comparado à fração de ejeção do ventrículo esquerdo, o strain longitudinal global (SLG) é uma avaliação mais precisa da função sistólica, importante em diversas situações, como cardio-oncologia,^2^ doença cardíaca valvar,^3^ cardiomiopatias^4^ e doença cardíaca isquêmica.^5^ Trata-se de um dado mais preciso e reprodutível, intimamente correlacionado com o prognóstico e atualmente disponível na maioria dos aparelhos de ecocardiograma.^6^ Por outro lado, essa análise requer uma janela acústica adequada, e a questão da variabilidade entre as empresass ainda deve ser levada em consideração.^7^

Um passo muito significativo que trouxe a análise da deformação miocárdica para a nossa realidade brasileira foi dado em 2023 com a publicação do “ Posicionamento do Departamento de Imagem Cardiovascular da Sociedade Brasileira de Cardiologia sobre o Uso do Strain Miocárdico na Rotina do Cardiologista – 2023” no periódico Arquivos Brasileiros de Cardiologia.^8^ Diversos especialistas se uniram na produção deste importante documento que ampliou o conhecimento dos cardiologistas sobre o SLG. Publicado na edição atual dos Arquivos Brasileiros de Cardiologia, mais um passo foi dado com o estudo ELSA-Brasil. O ELSA-Brasil é um amplo estudo epidemiológico com mais de 15.000 servidores públicos de universidades e instituições de pesquisa sobre doenças cardiovasculares e diabetes, realizado em 6 cidades brasileiras (São Paulo, Rio de Janeiro, Belo Horizonte, Vitória, Salvador e Porto Alegre), com diversas publicações na literatura.^9^ Desta vez, o foco foi a determinação dos valores de normalidade para o strain longitudinal global do ventrículo esquerdo (SLGVE) e o strain longitudinal da parede livre do ventrículo direito (SLPLVD), ou seja, uma investigação da deformação miocárdica em nossa população.

Em 2019, o estudo WASE (*World Alliance Societies of Echocardiography*) para valores normais, que envolveu 19 centros em 15 países, representando 6 continentes, incluiu 2.008 indivíduos com os principais objetivos de estabelecer valores normais para o tamanho do ventrículo esquerdo e função sistólica em uma população distribuída mundialmente, com diferentes nacionalidades, raças e etnias.^10^ Foi observada uma variação pequena, mas estatisticamente significativa, no SLGVE. A ideia inicial deste estudo baseou-se no fato de que os valores normais utilizados mundialmente provêm de estudos realizados nos Estados Unidos e na Europa com populações dessas localidades.^11^

Considerando as diferentes características demográficas das populações em diferentes regiões do mundo, faz todo o sentido analisar a deformação miocárdica especificamente em nossa população. Seguindo essa premissa, o ELSA-Brasil incluiu um total de 1.048 indivíduos entre agosto de 2008 e dezembro de 2010. Vale ressaltar que, desse total, para obter valores normais de SLGVE e SLPLVD foi utilizado um “filtro para características patológicas” que sabidamente alteram a deformação miocárdica, como hipertensão, diabetes, obesidade e doença renal. Assim, foi analisada uma subamostra de 527 participantes considerados “saudáveis”, com média de idade de 50,2 anos e 59% do sexo feminino. A média de SLGVE foi de 19,0% (14,3-23,8) e SLPLVD foi de 28,3% (22,3-34,3). Mulheres apresentaram maior SLGVE (19,5 ± 2,3 [15-24]) do que homens (18,3 ± 2,3 [14-23]), sem diferenças significativas relacionadas à idade. Valores mais elevados de SLGVE em mulheres também foram demonstrados no WASE, bem como nos estudos NORRE e HUNT.

O estudo NORRE (*Normal Reference Ranges for Echocardiography*) foi realizado na Europa e contou com um total de 22 centros participantes. Este estudo incluiu 549 indivíduos saudáveis (idade média: 45,6 ± 13,3 anos). Eles observaram um valor médio normal de SLGVE de 22,5% ± 2,7 (17,2-27,7), sendo maior em mulheres (23,0% ± 2,7 [17,8-28,2]) em comparação aos homens (21,7% ± 2,5 [16,7-26,7]).^12^

O estudo HUNT é um amplo estudo norueguês semelhante ao ELSA-Brasil, que há anos investiga diversos parâmetros normais em uma população do Condado de Trøndelag. Incluiu 1.266 indivíduos e também demonstraram valores mais elevados em mulheres (17,4% ± 4,6) em comparação aos homens (15,9% ± 4,6).^13^ O estudo mais recente HUNT4-Echo avaliou 2.462 indivíduos entre 2017 e 2018. Eles descreveram um valor normal para SLGVE de 20% (16-24) e para SLPLVD de 25,9% (17,4%-34,5).^14^

Um ponto que vale destacar é que o SLGVE obtido no estudo ELSA-Brasil foi derivado da análise de 12 segmentos, apenas dos cortes de 4 e 2 câmaras, rotineiramente adquiridos naquela época. Isso deve ser levado em consideração, pois pode ser uma limitação, fato reconhecido pelos autores. Valores anormais de SLGVE, definidos como < 14%, foram encontrados em 3,8% da população, associados à obesidade, hipertensão e diabetes. Valores anormais de SLPLVD, definidos como < 22%, correlacionaram-se com obesidade e aumento da massa ventricular esquerda.

Sobre a evolução do SLGVE com o envelhecimento, todos esses grandes estudos internacionais demonstraram uma redução na deformação miocárdica com a idade. O ELSA-Brasil não demonstrou isso e um dos motivos seria a estreita faixa etária da amostra, conforme apontado pelos os autores.

Concluindo, é muito comum extrair valores de normalidade de diversos estudos internacionais. No entanto, é importante ressaltar que, em parâmetros sensíveis a aspectos demográficos, como a deformação miocárdica, dados da população local são importantes. Isso foi feito com elegância pelo estudo ELSA-Brasil, e os autores devem ser reconhecidos pela publicação.^15^
