# Supplementary material for: O Estudo ELSA-Brasil e Nossa Deformação Miocárdica
Source: Arq Bras Cardiol. 2025 Jun 4;122(6):e20250265. [Article in Portuguese] doi: 10.36660/abc.20250265 (PMC12212377; doi:10.36660/abc.20250265)
Supplement: Translation

# Translation: The ELSA-Brasil Study and Our Myocardial Deformation

**DOI:** 10.36660/abc.20250265i

**Authors:** Márcio S. M. Lima

**Affiliations:** 1 Brazil Instituto do Coração do Hospital das Clínicas da Faculdade de Medicina da Universidade de São Paulo, São Paulo, SP – Brazil; 2 Brazil Grupo Fleury, São Paulo, SP – Brazil

**Keywords:** Echocardiography, Ventricular Function, Longitudinal Studies

The analysis of myocardial deformation (strain) using speckle tracking technology in echocardiography is no longer something new and innovative. Several years have passed since its validation.^1^ Compared to left ventricular (LV) ejection fraction, global longitudinal strain (GLS) is a more accurate assessment of systolic function, important in several situations, such as cardio-oncology,^2^ valvular heart disease,^3^ cardiomyopathies,^4^ and ischemic heart disease.^5^ It is more accurate and reproducible data, is closely correlated with prognosis, and is currently available in most echocardiogram machines.^6^ On the other hand, this analysis requires an adequate acoustic window, and the issue of inter-vendor variability must still be taken into account.^7^

A highly significant step that brought the analysis of myocardial deformation to our Brazilian reality was taken in 2023 with the publication of the “Position Statement on the Use of Myocardial Strain in Cardiology Routines by the Brazilian Society of Cardiology’s Department Of Cardiovascular Imaging – 2023” in the *Arquivos Brasileiros de Cardiologia* journal.^8^ Several experts joined in the production of this important document that increased cardiologists’ knowledge about GLS. Published in the current issue of the *Arquivos Brasileiros de Cardiologia*, another step was taken through the ELSA-Brasil study. ELSA-Brasil is a broad epidemiological study with more than 15,000 public servants from universities and research institutions on cardiovascular diseases and diabetes, carried out in 6 Brazilian cities (São Paulo, Rio de Janeiro, Belo Horizonte, Vitória, Salvador, and Porto Alegre), with several publications in the literature.^9^ This time, the focus was on determining normal values for LVGLS and right ventricular free wall longitudinal strain (RVFWLS), that is, an investigation of myocardial deformation in our population.

In 2019, the WASE (World Alliance Societies of Echocardiography) study for normal values, which involved 19 centers in 15 countries representing 6 continents, included 2,008 individuals with the main objectives of establishing normal values for LV size and systolic function in a population distributed worldwide, with different nationalities, races, and ethnicities.^10^ A small but statistically significant variation in LVGLS was observed. The initial idea for this study was based on the fact that the normal values used worldwide come from studies carried out in the United States and Europe with populations from these locations.^11^

Considering the different demographic characteristics of populations in different regions around the world, it makes perfect sense to analyze myocardial deformation specifically in our population. Following this premise, ELSA-Brazil included a total of 1,048 individuals from August 2008 to December 2010. It is worth noting that of this total, in order to obtain normal values for GLS and RVFWLS, a “filter for pathological characteristics” that are known to alter myocardial deformation, such as hypertension, diabetes, obesity, and kidney disease, was used. Thus, a subsample of 527 participants considered “healthy”, with a mean age of 50.2 years and 59% female, was analyzed. The mean LVGLS was 19.0% (14.3-23.8), and RVFWLS was 28.3% (22.3-34.3). Females exhibited higher LVGLS (19.5 ± 2.3 [15-24]) than males (18.3 ± 2.3 [14-23]), with no significant age-related differences. Higher LVGLS values in females were also demonstrated in the WASE, as well as in the NORRE and HUNT studies.

The NORRE (Normal Reference Ranges for Echocardiography) study was conducted in Europe and had a total of 22 participating centers. This study included 549 healthy individuals (mean age: 45.6 ± 13.3y). They observed a mean normal GLS value of 22.5% ± 2.7 (17.2-27.7), being higher in women (23.0% ± 2.7 [17.8-28.2]) compared to men (21.7% ± 2.5 [16.7-26.7]).^12^

The HUNT study is a large Norwegian study similar to ELSA-Brasil, which has been investigating several normal parameters in a population from Trøndelag County for years. It included 1,266 individuals and also demonstrated higher values in women (17.4% ± 4.6) compared to men (15.9% ± 4.6).^13^ The more recent HUNT4-Echo study evaluated 2,462 individuals between 2017 and 2018. They described a normal value for LVGLS of 20% (16-24) and for RVFWLS of 25.9% (17.4%-34.5).^14^

One point worth highlighting is that the LVGLS obtained in the ELSA-Brasil study was derived from analyses of 12 segments, only from the 4- and 2-chamber views, which were routinely acquired at that time. This should be taken into account because this may be a limitation, a fact acknowledged by the authors. Abnormal GLS values, defined as < 14%, were found in 3.8% of the population, associated with obesity, hypertension, and diabetes. Abnormal RVFWLS values, defined as < 22%, were correlated with obesity and increased LV mass.

Regarding the evolution of LVGLS with aging, all these large international studies have demonstrated a reduction in myocardial deformation with age. ELSA-Brazil did not demonstrate this. As the authors pointed out, one reason here would be the narrow range of the sample.

In conclusion, it is very common to extract values of normality from various international studies. However, it is important to emphasize that in parameters that are sensitive to demographic aspects, such as myocardial deformation, data from the local population are important. This was done elegantly by the ELSA-Brasil study, and the authors should be acknowledged for the publication.^15^
